# PReS-FINAL-2227: QT and JT dispersion in children with FMF

**DOI:** 10.1186/1546-0096-11-S2-P217

**Published:** 2013-12-05

**Authors:** K Fidanci, A Kilic, M Gulgun, C Acikel, G Basbozkurt, E Demirkaya, F Gok

**Affiliations:** 1 Gulhane Military Medical Academy, Ankara, Turkey

## Introduction

Familial Mediterranean Fever (FMF) is an autoimmune, autosomal recessive inherited disorder, and characterized by recurrent episodes of peritonitis, plöritis and arthritis. Patients with inflammatory disease are at increased risk of cardiovascular complications due to rhythm disorders. QT and JT dispersions are simple and non-invasive arrhythmogenic markers and can be used to assess the homogeneity of cardiac repolarization.

## Objectives

The aim of this study was to determine the risk of cardiac arrhythmias in patients with FMF by evaluating QT and JT dispersion.

## Methods

The study group and the control group were evaluated with a standard 12-lead electrocardiography (ECG). QT, JT and RR distances were measured in both groups. The corrected QT (QTc) and corrected JT (JTc) were calculated. QT dispersion (QTcd) and JTd dispersion (JTcd) were determined.

## Results

A total of 48 FMF patients who are in the attack-free period and use regular colchicine therapy (26 male, 22 female, 11.10 ± 3.42 years) and 31 healthy children (17 males, 14 females, 9.61 ± 2.83 years) were included in the study.

There was no statistically significant difference was found between the study and control groups in terms of RR, QT, QT, QTcd, JT, JTc, JTd and JTcd measurements. QTc value is found to be higher in patients with FMF than the control group (412.15 ± 21.45-393.58 ± 35.18, t = 2916, p = 0.005), although the difference was statistically significant, the value is within normal limits (below 0.44).

## Conclusion

In our study it is tried to determine the risk factor by investigation of QT and JT time and dispersion. QTc value is higher than in patients with FMF than the control group. There is no significant difference in QT and JT dispersion between the groups but the prolonged QTc value may increase the risk of arrhythmia as the indicator of ventricular sensitivity.

QTc value shows the increase in ventricular sensitivity and is an important indicator of cardiovascular mortality. It would be useful to monitor FMF patients with electrocardiography because of the risk of sudden cardiac death.

## Disclosure of interest

None declared.

